# Role of Circulating Tumor DNA in Adapting Immunotherapy Approaches in Breast Cancer

**DOI:** 10.3390/curroncol32070373

**Published:** 2025-06-26

**Authors:** Sudhir Kumar, Rossanna C. Pezo

**Affiliations:** Division of Medical Oncology and Hematology, Department of Medicine, Sunnybrook Health Sciences Centre, University of Toronto, 2075 Bayview Avenue, Toronto, ON M4N 3M5, Canada; sudhir.kumar@sunnybrook.ca

**Keywords:** immunotherapy, breast cancer, pembrolizumab, circulating tumor DNA

## Abstract

Immunotherapy has a defined role in the treatment of both early- and late-stage triple-negative breast cancer (TNBC) and is under active exploration in human epidermal receptor 2-positive as well as high-risk hormone-receptor-positive subtypes. It is critical to balance the efficacy and toxicity of immunotherapy while keeping the cost and duration of treatment in check. In addition to the immunohistochemistry testing of PD-L1 expression, which only predicts the efficacy of immunotherapy in metastatic TNBC, there is a lack of biomarkers that are better standardized to predict efficacy and treatment response, detect early relapse, and guide prognosis in breast cancer patients treated with immunotherapy. Circulating tumor DNA (ctDNA) is a minimally invasive, dynamic, real-time, blood-based biomarker that has shown promising value in the management of solid tumors, including breast cancer. This review discusses the emerging evidence for the potential application of ctDNA to further refine patient-centered care and personalize treatment based on a molecularly defined risk assessment for breast cancer patients treated with immunotherapy-based approaches. We further discuss the challenges and barriers to widespread adoption of this promising tool in the management of breast cancer patients requiring immunotherapy.

## Simple Summary

The role of immunotherapy is well defined in treating metastatic or early-stage triple-negative breast cancer (TNBC) and is being actively studied in other breast cancer types. While effective, immunotherapy can come with side effects and high costs, making it important to individualise treatment. Currently, PD-L1 testing is the only approved method to predict immunotherapy response, and only in metastatic TNBC. However, there is a need for better biomarkers to predict treatment outcomes, detect relapse early, and guide prognosis across various types of breast cancer. Circulating tumor DNA (ctDNA), a blood-based biomarker, offers a promising and minimally invasive way to monitor disease and tailor treatment in real-time. It has shown value in managing various solid tumors, including breast cancer. This review explores how ctDNA could play a role in personalized care for breast cancer patients receiving immunotherapy and highlights the challenges that need to be addressed to maximize its clinical utility.

## 1. Introduction

Anti-programmed cell death receptor 1 (PD-1) agent-based combination therapy is the current standard first-line treatment in early-stage triple-negative breast cancer (TNBC) and PD-L1 + metastatic TNBC as it significantly improves clinical outcomes. More recently, the addition of nivolumab (anti-PD-1) or pembrolizumab (anti-PD-1) to neoadjuvant chemotherapy in high-risk, hormone-receptor-positive (HR+) breast cancer (BC) has been shown to improve pathological complete response (pCR) rates, although the data on survival benefits are immature at present [1,2]. The role of immunotherapy in human epidermal receptor 2-positive (HER2+) subtypes is an area of active investigation.

The assessment of tumor PD-L1 expression by immunohistochemistry (IHC) predicts the efficacy of pembrolizumab in metastatic TNBC, while patients with higher levels of stromal tumor-infiltrating lymphocyte levels or PD-L1 expression derive benefit from nivolumab in early-stage HR + BC [1,3]. While benefit from immunotherapy is limited to a subset of patients with metastatic TNBC or high-risk HR + BC, any curative intent treatment with immunotherapy warrants a fine balance of efficacy and side effects. Regardless of the stage of BC, cost considerations and duration of treatment with immunotherapy have implications for quality of life and toxicity. Thus, it is crucial to identify markers that predict efficacy, monitor response to treatment, detect early relapse, guide prognosis, and help improve the personalization of BC treatment with immunotherapy.

Circulating tumor DNA (ctDNA) is a minimally invasive, real-time, blood-based biomarker that has shown promise in diagnosis, prognosis, and predicting therapy response in various cancers over the last decade. Society guidelines currently recommend genomic testing using next-generation sequencing (NGS) technologies on patient’s plasma through ctDNA to identify actionable targetable mutations or emergent resistance alteration in advanced solid tumors including BC (e.g., PIK3CA or ESR1 mutations) [4,5]. Among non-metastatic solid tumors treated with neoadjuvant immunotherapy, a lack of ctDNA clearance may identify patients unlikely to achieve a pathological complete response [6,7]. In early breast cancer treated with non-immunotherapy approaches, baseline ctDNA positivity portends a worse prognosis, and an inability to clear ctDNA during neoadjuvant chemotherapy or adjuvant treatment reflects treatment resistance and an increased risk of metastatic recurrence [8].

In this review, we discuss the key evidence for the role of ctDNA in various avenues of breast cancer treatment with immunotherapy at present. We also delineate the challenges in the application of ctDNA in the individualization of immunotherapy approaches in breast cancer.

### 1.1. Approach to ctDNA Detection and Interpretation

ctDNA includes small pieces of DNA released into the blood stream from tumor deposits or circulating tumor cells and is characterized by a half-life of up to 2 h [9]. Even though ctDNA has a lower concentration in plasma, it is preferred over serum for estimation to avoid contamination from DNA derived from normal cells [10]. The ctDNA assays may employ a non-targeted approach in which the whole genome or exome of ctDNA is analysed. Otherwise, a targeted approach that focuses on a predetermined set of genes may also be used. The targeted approach can be tumor-naïve (predefined selected genes) or tumor-informed (genes previously detected in tumor tissue) [11,12]. Examples of commercially available tumor-agnostic ctDNA tests in breast cancer include Guardant Reveal, FoundationOne Liquid CDx, and Guardant 360 CDx. Similarly, the common available tumor-informed assays are Signatera, Invitae personalised cancer monitoring, Haystack MRD, and RaDaR. Although the non-targeted approaches provide a more global mutational landscape of the tumor, they are limited by low sensitivity, low specificity and higher cost [13,14]. The older techniques like quantitative polymerase chain reaction (qPCR) have been superseded by modern techniques like droplet digital PCR (ddPCR) due to higher sensitivity, albeit at the cost of being more labour intensive. Other targeted DNA sequencing techniques include tagged amplicon deep sequencing (TAM-Seq), cancer personalized profiling by deep sequencing (CAPP-Seq), safe sequencing system (Safe-Seq), and amplicon sequencing (AmpliSeq). Next-generation sequencing is now the preferred approach, as it can interrogate whole genomes or many genes as compared to ddPCR, which assesses only a small number of alterations in a single experiment. Variant allele frequency (VAF) reported by an NGS test is the percentage of mutant sequence reads divided by overall coverage at that particular focus. The most modern ultrasensitive sequencing methods can detect VAF as low as 0.02% at a specificity of more than 95 percent [15].

Being a blood-based biomarker, ctDNA offers various advantages over tissue-based tests, including minimal invasiveness, ease of serial testing, rapid turnaround time and reproducibility [16]. Moreover, it promises to better capture the tumor heterogeneity and temporal spacing of the tumor genotype. When compared to the testing of other liquid biopsy components like circulating tumor cells (CTCs) or exosomes, ctDNA assessment methods are more mature and better established. Additionally, ctDNA allows for the detection of tumor heterogeneity and blood-based tumor mutational burden, which the other liquid biopsy components cannot assess [17].

**Figure 1:**
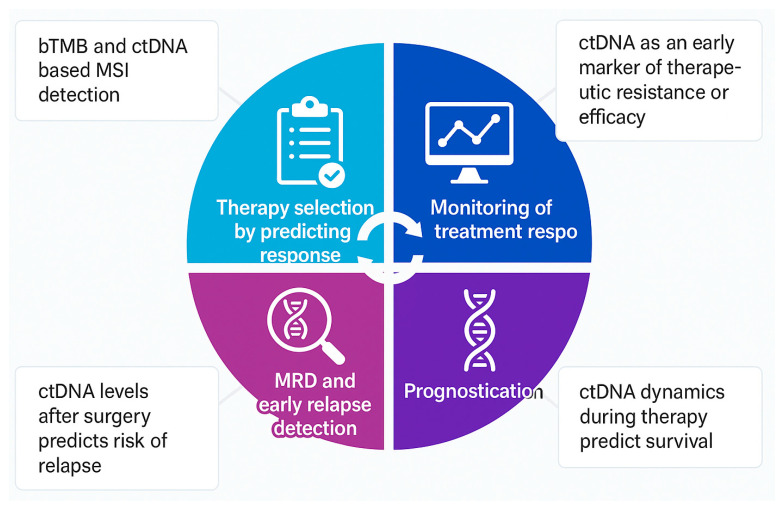
Overview of potential ctDNA applications in breast cancer treated with immunotherapy. bTMB: Blood-based tumor mutational burden; MSI: microsatellite instability; ctDNA: circulating tumor DNA.

The validation of ctDNA as a biomarker in breast cancer patients treated with immunotherapy necessitates a demonstration of analytical validity (technical accuracy and reliability in ctDNA detection), clinical validity (ability to stratify clinically meaningful subgroups based on predictive or prognostic relevance), and clinical utility (inform therapeutic decision making leading to measurable improvement in outcomes [18]. Nevertheless, high cost, CHIP (clonal hematopoiesis of indeterminate potential)-induced false positivity, variable sensitivity of the assays depending upon the number of tumor specific variants or timepoints, and false negative results due to the low fraction of detectable ctDNA are important barriers in the widespread application of this biomarker in oncology [19,20]. Key large prospective studies evaluating the role of ctDNA in breast cancer treated with immunotherapy are summarised in Table 1. An overview of the potential areas of ctDNA utility in this setting is shown in Figure 1.

### 1.2. Predicting Response to Immunotherapy

Prospective evidence suggests that ctDNA can be detected in patients with metastatic breast cancer with approximately 80% sensitivity and specificity, which is further improved by longitudinal testing [22]. Given multiple tissue-specific and tissue-agnostic indications for targeted therapy, NGS testing via ctDNA is now one of the standards in advanced breast cancer [4,5]. Baseline genomic instability and ctDNA concentration have been shown to predict responses to immune check point inhibitors (ICI) [23]. Genomic biomarkers like tumor mutational burden (TMB) and microsatellite instability (MSI) may help predict responses to ICIs in advanced solid tumors including breast cancer. Based on the results of the Keynote-158 study, pembrolizumab monotherapy received accelerated FDA approval for unresectable or metastatic solid tumors with TMB ≥ 10 mutations per megabase. In this non-randomised phase 2 study, 13% (*n* = 102) of enrolled patients had high TMB, spread across 27 different tumor types [24]. Tissue TMB (tTMB) was assessed win formalin-fixed, paraffin-embedded tumor samples using the FoundationOne CDx assay. The objective response rate (complete or partial response using response evaluation criteria in solid tumors, version 1.1) observed in those with high tTMB was 29% as compared to 6% in the non-high-TMB group. High tTMB is observed in 5% of breast cancers overall, with varying incidence based on tumor subtype (TNBC > HR−/HER2+ > HR+/HER2−) and sample site (metastatic > primary). The quantification of TMB can alternatively be obtained from blood samples using ctDNA. While the detection of MSI status in ctDNA has been adequately validated and can be used for treatment selection, there is currently limited evidence that blood TMB alone can be used for treatment with pembrolizumab [25,26]. Initial data from a large study of approximately 4000 breast cancer patients that employed tumor whole genome sequencing shows that hypermutation is a predictor of benefit from PD-1 inhibitors [27]. Despite the FDA’s tissue-agnostic approval for high TMB (≥10 mutations per DNA mega base pair), the proportion of breast cancer patients meeting this threshold varies with tumor subtype. Although still investigational, TMB assessment from ctDNA may be an alternative to tissue-based estimation in those with limited tumor tissue or patients who are not good candidates for re-biopsy, as there is a statistically significant correlation between blood-based TMB and tissue-based TMB [28]. Of note, the optimal TMB cutoff in predicting benefit to ICI treatment in advanced breast cancer is yet to be established [29].

The deficiency in DNA mismatch repair leads to microsatellite instability, a phenomenon that has been seen in various tumors, including breast cancer. Owing to the improved response of any dMMR/MSI tumor to PD-1/PD-L1 blockade, pembrolizumab received tissue-agnostic approval from the FDA in this space [30]. This approval was based on clinically meaningful response rates observed in patients with MSI-H/dMMR enrolled across five clinical trials. The MSI-H or dMMR status in tumors was prospectively assessed using PCR or IHC tests. Among 149 patients with MSI-H/dMMR tumors, the overall response rate was 39.6%, with 7% complete responses. Utilising dMMR as a predictive marker may guide immune therapy selection in advanced breast cancer, although the prevalence of dMMR/MSI breast cancer remains low (<2%) [31]. Regardless, high MSI can also be detected using ctDNA-based essays even at low coverage [32].

The Keynote-522 trial showed that neoadjuvant pembrolizumab with chemotherapy followed by adjuvant pembrolizumab significantly improved pCR, EFS, and overall survival (OS) vs. neoadjuvant placebo plus chemotherapy with adjuvant placebo in patients with high-risk early TNBC [33]. This led to the establishment of chemoimmunotherapy as standard neoadjuvant therapy in stage 2–3 TNBC. The benefits of chemoimmunotherapy were seen regardless of PD-L1 status, and ctDNA was not one of the biomarkers studied in this seminal trial. To date, no predictive biomarkers of chemoimmunotherapy response have been identified in this space.

In summary, while ctDNA has become a standard technique to predict therapeutic response in breast cancer treated with targeted therapies based on mutational signature, a similar clinical utility is currently limited to a fraction of advanced breast cancer patients treated with immunotherapy.

### 1.3. Monitoring Response to Immunotherapy

The traditional methodologies for assessing treatment response in breast cancer have been clinical examination, pathological examination, various imaging modalities, and sometimes non-specific blood-based biomarkers. Pathological examination post neoadjuvant systemic therapy and surgery remains the gold standard for tumor response assessment. These approaches, while useful, are often limited by their relative lack of sensitivity. Moreover, in the current era of targeted therapies and immunotherapies, there is an urgent need to obtain detailed molecular insights which are crucial for clinical decision making. Liquid biopsy, especially ctDNA, has emerged as a dynamic platform for the real-time analysis of cancer burden and molecular signatures conferring treatment resistance.

The ease of testing with a blood-based biomarker like ctDNA allows serial monitoring of treatment response and, given evidence that changes in ctDNA precede clinical or radiographic response, this modality offers potential utility as an early marker of therapeutic resistance or efficacy. INSPIRE was a phase 2 trial that provided evidence for ctDNA-based monitoring in breast cancer treated with ICI [34]. One of the aims of this study was to prospectively investigate whether early changes in ctDNA levels would precede radiological response in multiple cohorts of advanced solid tumors treated with pembrolizumab. This study enrolled 18 patients with metastatic TNBC among a total of 106 and performed serial personalized ctDNA assessments at baseline and before the third cycle of treatment. An early reduction in ctDNA after two cycles of pembrolizumab was associated with significant clinical benefit regardless of tumor subtype, PD-L1 status or TMB. On treatment, ctDNA clearance also showed a similar correlation. Moreover, an early rise in ctDNA levels was associated with significantly higher rates of disease progression. These early ctDNA dynamics were also complementary to imaging criteria for identifying patients with no response and poor outcomes. Larger studies are needed to test the utility of ctDNA in distinguishing true progression from pseudoprogression early during immunotherapy. Similarly, future studies are needed to examine the role of ctDNA in identifying breast cancer patients who may benefit from continuing immunotherapy beyond progression.

In stage 2–3 breast cancer treated with neoadjuvant chemotherapy, ctDNA has demonstrated its utility as a robust predictor of pathological response and disease recurrence [8]. Also, recently developed predictive models for neoadjuvant chemotherapy that combine ctDNA with genomic and clinical features have successfully predicted pathological response and survival outcomes in breast cancer patients [35]. In non-metastatic breast cancer, the addition of pembrolizumab to neoadjuvant chemotherapy was studied in the I-SPY2 trial, and one of the endpoints was to investigate the association of ctDNA with pCR using a personalized ctDNA assay [36]. Among 511 samples collected longitudinally from 138 patients, the early clearance of ctDNA during NAC treatment was significantly associated with an increased likelihood of achieving pCR. In a meta-analysis of 13 trials involving 380 patients diagnosed with various solid tumors and treated with neoadjuvant ICI, ctDNA clearance before surgery was 98% sensitive for predicting pCR, but the specificity of ctDNA persistence for a lack of pCR was 53%, indicating that half of the patients had residual disease after ctDNA clearance after neoadjuvant therapy [21]. High heterogeneity and low specificity in this meta-analysis warrant a cautious approach in relying on ctDNA clearance to inform treatment decisions in the neoadjuvant setting. Monitoring ICI response with ctDNA is limited in tumors with no or low levels of ctDNA shedding or those restricted to protected compartments like the central nervous system. Moreover, the interpretation of serial ctDNA levels is complicated by intercurrent infection or inflammation, time of collection, and sample handling and processing [37].

### 1.4. Minimal Residual Disease (MRD) and Early Relapse Detection

Traditionally, after neoadjuvant systemic therapy, pCR has been one of the key markers to assess treatment response, guide subsequent therapeutic choices, and personalize intensity or duration of treatment in breast cancer. Detectable ctDNA has a short half-life, and its persistence after curative surgery indicates persistent or recurrent disease and is strongly predictive of recurrence in real time. The assessment of MRD with ctDNA after surgery in patients treated with neoadjuvant systemic therapy could provide significant clinical value, including the identification of patients at high risk of relapse, thus allowing tailored treatment to maximize benefits while minimizing toxicity. The enhanced sensitivity of new ctDNA assays using any of the tumor-informed or tumor-agnostic approaches now allows for the detection of very low levels of ctDNA, <0.01% mutant allele fraction, which is expected to improve the predictive efficacy of MRD clearance in relation to pCR.

Prospective studies employing tumor-informed approaches and/or NGS-based ctDNA assessment in patients with breast cancer treated with neoadjuvant chemotherapy have shown that the detection of MRD after neoadjuvant treatment was associated with future clinical relapse, inferior disease-free survival, and overall survival [38,39]. The I-SPY2 trial showed that the presence of ctDNA after neoadjuvant immunotherapy has been associated with lower pCR rates, while the clearance of ctDNA after treatment was associated with longer survival even in patients who did not achieve a pCR [8]. The c-TRAK TN trial prospectively evaluated the utility of ctDNA surveillance in patients with TNBC with MRD-positive disease and no imaging evidence of metastasis who received pembrolizumab [40]. One of the primary end points of this study was to validate whether ctDNA-based MRD could be detected with sufficient lead time bias before radiographic evidence of disease to affect clinical management. The trial did not meet this end point. It still confirmed that early ctDNA detection was associated with high rates of metastatic disease and highlighted the need to have more frequent timepoints and higher sensitivity assays in ctDNA detection. Currently, there is insufficient evidence on whether MRD detection in breast cancer can guide therapy to improve clinical outcomes. ASPRIA (NCT04434040) is a phase 2 trial investigating whether intensified adjuvant treatment with a combination of immunotherapy and antibody drug conjugate can eliminate ctDNA and improve survival outcomes in ctDNA-positive patients with residual disease following curative surgery.

One of the key limitations of ctDNA-based MRD evaluation in breast cancer is the highly variable sensitivity, albeit with a consistently high specificity of current assays, which indicates that a negative MRD test does not reliably predict whether a patient will experience recurrence. Overall, the available literature promises ctDNA as a standalone, convenient, and early marker to detect therapy resistance and predict early recurrence in breast cancer treated with immunotherapy. Large clinical trials with long-term follow ups are required to prove the efficacy of changing therapy in response to ctDNA dynamics.

### 1.5. Assessment of Prognosis

Prospective and real-world studies in advanced breast cancer treated with non-immunotherapy approaches have demonstrated the prognostic value of ctDNA. For example, higher baseline ctDNA levels have been associated with an increased risk of progression, while serial decreases in ctDNA levels during treatment have been shown to correlate with better PFS and OS when patients were treated with cyclin D kinase 4/6 inhibitors [41,42]. In addition, a higher baseline ctDNA fraction of >10% was independently associated with worse outcomes in metastatic TNBC treated with chemotherapy [43]. The INSPIRE trial mentioned above prospectively assessed ctDNA dynamics as a biomarker of tumor burden in diverse advanced cancer patients treated with pembrolizumab [34]. As compared with the baseline, lower ctDNA levels before the third cycle of pembrolizumab were associated with more favourable PFS and OS, while rising ctDNA levels correlated with a poor median OS of 13.7 months.

In early-stage breast cancer, ctDNA analysis is difficult due to the lower ctDNA concentration compared with metastatic disease and differences in the rate of ctDNA shedding based on the type of BC; luminal BC shedding has lower levels compared with HER2 + BC and TNBC. Nevertheless, the evidence for a prognostic role for ctDNA in this setting continues to evolve due to advances in sequencing and bioinformatics.

In the MONARCH E trial that treated HR+ HER2− early BC with adjuvant abemaciclib for two years, persistent ctDNA positivity was associated with more invasive disease-free survival events versus those with continued negativity of ctDNA status [44]. In other early-stage cancers treated with immunotherapy, ctDNA clearance has been shown to be predictive of pCR and improved survival [6,21]. Specifically in breast cancer, the I-SPY2 trial mentioned above showed that all patients with early-stage disease (*n* = 34) who cleared ctDNA before surgery had pCR. Among those who failed to achieve pCR, distant recurrence-free survival was significantly better in patients who cleared ctDNA before surgery compared to those who had positive ctDNA status [36]. The exploratory analysis data from the phase 3 KEYNOTE-522 study was presented at the San Antonio Breast Cancer Symposium in December 2024. Improvements in pCR and EFS with or without pembrolizumab were associated with biomarkers such as T-cell-inflamed 18-gene expression profiles, but ctDNA assessments were not part of this study. TMB was also correlated with improved EFS in the pembrolizumab plus chemotherapy arm (*p* ≤ 0.001), but not in the placebo plus chemotherapy arm (*p* = 0.011 for pCR; *p* = 0.422 for EFS). There were few TNBC patients with high TMB in this patient population. While ctDNA can be a real-time, non-invasive strategy to predict long-term survival, future large prospective studies are needed to further confirm its role.

### 1.6. Current Challenges

Despite the strong promise of ctDNA as a tool for adapting immunotherapy approaches in breast cancer, several challenges remain. To begin with, there is a lack of a harmonized threshold for risk stratification at baseline, along with issues pertaining to diverse detection approaches, with heterogenous coverage, fragmentation sizes, and thresholds of detection. Over the last half a decade, advancements in technology have allowed for the detection of various genomic alterations via ctDNA in oncology with higher sensitivity. However, a lower concentration of ctDNA as compared to other liquid biopsy components, coupled with variable shedding based on the stage of BC, type of BC, and sites of disease, limits its detection. Moreover, the sensitivity of current ctDNA assays is suboptimal, especially in early-stage disease, with a negative predictive value less than 80%. Emerging techniques like low-pass whole-genome sequencing (LP-WGS) may help overcome technical barriers in detecting scarce ctDNA levels in breast cancer patients. LP-WGS allows for the detection of rare ctDNA fragments by providing broad genomic coverage at a lower depth, thereby improving sensitivity.

There is also a lack of guidelines on the standardization of pre-analytical steps like collection or the processing of samples and downstream analytical assays used, which has often led to significant heterogeneity in clinical trial methodology and results.

Compared with tissue-based biomarkers, dynamic real-time ctDNA assessments promise the better capture of spatial and temporal tumoral heterogeneity, but the optimum frequency and timing of ctDNA testing is currently unknown. The integration of clinical features, radiomic properties, and ctDNA analysis may fully recapitulate tumor heterogeneity in the future. Also, the modern methods of liquid biopsy, like ultra deep sequencing, are limited in their availability and/or affordability as these are expensive and bioinformatically challenging at present.

In advanced breast cancer treated with targeted approaches, there is a relatively large body of evidence for the clinical utility of ctDNA for predicting response, identifying resistance mechanisms, and the assessment of prognosis, so it is now the standard of care in this setting. On the other hand, in early-stage and in large subsets of advanced breast cancer treated with immunotherapy, the evidence on the clinical validity and clinical utility of ctDNA as a biomarker that can help direct therapeutic decisions is quite limited at present. Specifically, in breast cancer treated with curative intent approaches, long-term studies are needed to understand whether ctDNA-based MRD detection is actionable by clinically meaningful interventions. On the other hand, the evidence for a negative predictive value of ctDNA, which means that the absence of ctDNA does not equate to an absence of cancer, is practically non-existent. Currently, knowledge of ctDNA dynamics during immunotherapy without evidence of effective intervention can lead to more anxiety, invasive procedures, and frequent imaging, leading to a worse quality of life of patients with breast cancer.

## 2. Conclusions and Future Perspectives

As immunotherapy reshapes the treatment landscape of breast cancer, the search for elusive biomarkers that improve outcomes through a more personalized approach continues. The role of ctDNA in informing clinical decision making in breast cancer is increasing, with some applications already integrated into clinical care. Based on early signals from ctDNA dynamics in breast cancers treated with immunotherapy, further validation and implementation are required by means of prospective studies and well-designed clinical trials powered to assess patient-centric end points. Salient features of studies currently investigating the role of ctDNA in breast cancer treated with immunotherapy are shown in Table 2. The examination of ctDNA to identify patients who are at risk of immune-related adverse events is an unmet need in breast cancer patients. To improve its utility for patient selection and risk stratification, the integration of ctDNA with clinical and radiomic features and other liquid biopsy components is warranted.

## Figures and Tables

**Table 1:** Key studies of ctDNA analysis in breast cancer treated with immunotherapy.

Study	Design/Endpoints	Type of Breast Cancer	ctDNA Test	Key Findings
INSPIRE	Phase 2 study in five cohorts of advanced solid tumors treated with pembrolizumab	TNBC (*n* = 11) out of total 94 patients	Amplicon-based personalised bespoke ctDNA	Improved OS/PFS in patients with lowering or clearing of ctDNA on treatment
I-SPY2	Adaptive phase 2 clinical trial in high-risk stage 2–3 breast cancer testing with addition of multiple new agents to neoadjuvant chemotherapy including pembrolizumab	*N* = 138 (TNBC: 61, HR+/HER2−: 77)	Personalised ctDNA	ctDNA dynamics during neoadjuvant treatment predictive of pCR, metastatic recurrence, and death
Valenza et al. [21]	Meta-analysis of phase 1–3 clinical trials investigating ctDNA clearance and pCR in solid tumors treated with neoadjuvant immunotherapy	380 patients including breast cancer	Tumor-informed approach or tumor-naïve approach	Lack of ctDNA clearance may identify patients unlikely to have a pCR. The confirmatory power of ctDNA clearance is limited by low specificity and high heterogeneity due to the variability of the assays, and warrants further study
cTRAK-TN	Phase 2 clinical trial assessing utility of prospective ctDNA surveillance in TNBC and activity of pembrolizumab in patients with ctDNA+ residual disease	161 patients with TNBC	Personalized droplet PCR ctDNA assay	First study to assess the clinical utility of ctDNA in guiding therapy in TNBC; ctDNA detection associated with high risk of metastatic disease.

TNBC: Triple-negative breast cancer; OS: overall survival; PFS: progression-free survival; pCR: pathological complete remission; HR+: hormone receptor positive; HER2−: human epidermal growth factor receptor 2.

**Table 2:** Selected ongoing studies evaluating ctDNA-based immunotherapy adaptation in breast cancer.

Trial	Phase of Study	Stage and Type of Breast Cancer	Description	Role of ctDNA
APOLLO (NCT 04501523)	Phase 2 RCT	Early-stage TNBC	Patients treated with neoadjuvant chemotherapy and positive ctDNA after surgery are randomized to receive boost therapy (tislelizumab + capecitabine) vs. standard of care	ctDNA-based randomization
Artemis (NCT 04803539)	Phase 2 RCT	Early-stage TNBC	Investigates the benefit of boost therapy (Capecitabine + apatinib + camrelizumab) vs. standard of care (Capecitabine alone) in patients with positive ctDNA after surgery	ctDNA-based randomization
neoBREASTIM (NCT06067061)	Single-arm Phase 2	Early-stage TNBC	Evaluates novel, biomarker-driven combination of atezolizumab + RP1 oncolytic immunotherapy in neoadjuvant setting	ctDNA-based treatment continuation
PERSEVERE (NCT04849364)	Phase 2 RCT	Early-stage TNBC	Patients with residual disease after neoadjuvant treatment are assigned to one of the three arms	Along with a genomic biomarker assay, ctDNA is used as a biomarker for treatment selection
RESPONSE (NCT05020860)	Phase 2 parallel-arm	Early-stage TNBC	Study correlates early clinical response to pathological outcome with neoadjuvant systemic therapy	Correlation of ctDNA dynamics with clinical and pathological response
ASPRIA (NCT04434040)	Phase 2 single-arm	Early-stage TNBC	Study investigates the combination of atezolizumab + Sacituzumab govitecan in reducing recurrence risk in patients with residual disease and positive ctDNA in blood	ctDNA-based treatment selection
GIM 25 CAPT (NCT05266937)	Phase 2 single-arm	Metastatic TNBC	Studies combination of atezolizumab, paclitaxel, and carboplatin as first-line treatment in PDL-1-positive metastatic TNBC	Longitudinal ctDNA analysis during treatment
PAveMenT	Phase 1b	Androgen receptor-positive metastatic TNBC	Investigates combination of palbociclib + avelumab in this setting	Exploration of ctDNA as biomarker of response

ctDNA: Circulating tumor DNA; RCT: randomized controlled trial; TNBC: triple-negative breast cancer.
